# An Unusual Presentation of Gastric Variceal Hemorrhage

**DOI:** 10.7759/cureus.5406

**Published:** 2019-08-16

**Authors:** Divya Ravi, Fouzia Oza, Nishant Sharma, Bojana Milekic, Mahmoud Khalaf

**Affiliations:** 1 Internal Medicine, The Wright Center for Graduate Medical Education, Scranton, USA

**Keywords:** upper gi bleeding, endoscopy, varix, sensitivity, ct

## Abstract

Acute upper gastrointestinal (GI) bleeding is a commonly encountered condition that can potentially be life-threatening. Endoscopy is the diagnostic modality of choice, but it is important to recognize it's shortcomings. We introduce a 61-year-old female who presented with hematemesis and syncope. She had a history of recurrent episodes of hematemesis and hospitalizations for the preceding 18 months, for which multiple endoscopies had been performed but had failed to demonstrate a source. A repeat esophagogastroduodenoscopy (EGD) performed at our facility was unremarkable. A CT scan demonstrated a lobulated mass-like filling defect in the gastric cardia consistent with solitary varix with an abnormal fold pattern. An upper GI follow-through series was performed to better characterize this varix. The patient subsequently underwent balloon-occluded retrograde transvenous obliteration with successful control of the source of bleeding. It is important to keep in mind that EGD while being the gold standard for the diagnosis of varices, has its limitations, and should be augmented with the use of non-traditional diagnostic modalities such as CT scans or radionuclide imaging.

## Introduction

Acute gastrointestinal (GI) bleeding can be a life-threatening emergency and requires urgent medical attention. The incidence of upper GI bleeding, although declining in the United States, is still noteworthy, with an annual reported incidence of 60.6 per 100000 in 2009 [1]⁠. Historically, GI bleeding was divided into upper and lower GI bleeding [2]⁠. However, in recent years, GI bleeding has been characterized into upper GI bleeding (bleeding above the ampulla of Vater), middle GI bleeding (from the ampulla of Vater to the terminal ileum), and lower GI bleeding (bleeding beyond terminal ileum) [3]⁠.

Common sources of upper GI bleed include gastric and duodenal ulcers, esophageal varices, malignancy, and Mallory-Weiss tear [4]⁠. History and physical exam often provide crucial information that aide in narrowing down the differential towards a variceal versus a non-variceal source of bleeding.

## Case presentation

A 61-year-old woman presented with large volume hematemesis, abdominal pain, and syncope of acute onset. The syncopal episode was preceded by lightheadedness and precipitated by a change in position. Her past medical history was notable for non-insulin dependent diabetes mellitus, chronic obstructive pulmonary disease, and alcoholic cirrhosis Child-Pugh Class A, although she lacked any conspicuous signs of portal hypertension. Of note, the patient had multiple hospitalizations over the preceding year with similar presentations which were self-limiting, with no identifiable focus of bleeding. A review of her medical records revealed that she had undergone two esophagogastroduodenoscopies (EGD) over the prior 18 months; those reports were accessed, and while they did not specifically comment on the adequacy of visualization of the gastric anatomy, they reported an unremarkable esophagus, stomach, and duodenum.

Physical exam was notable for orthostatic hypotension and minimal epigastric tenderness. Laboratory workup was notable for normocytic anemia; her calculated Model of End-stage Liver Disease Serum Na (MELD-Na) score was 10. Early management included bowel rest, proton pump inhibitors, octreotide, and vigorous fluid resuscitation. She subsequently had multiple bowel movements productive of black tarry stools, along with worsening anemia requiring multiple blood transfusions. A repeat EGD was performed, and it showed a normal esophagus, stomach, and duodenum with no evidence of varices or active bleeding. A rectal exam was notable for a small amount of bright red blood mixed with the black tarry stools. A colonoscopy was performed to rule out a lower gastrointestinal (GI) bleed; it revealed a few small-mouthed non-bleeding diverticula with no other abnormalities.

**Figure 1:**
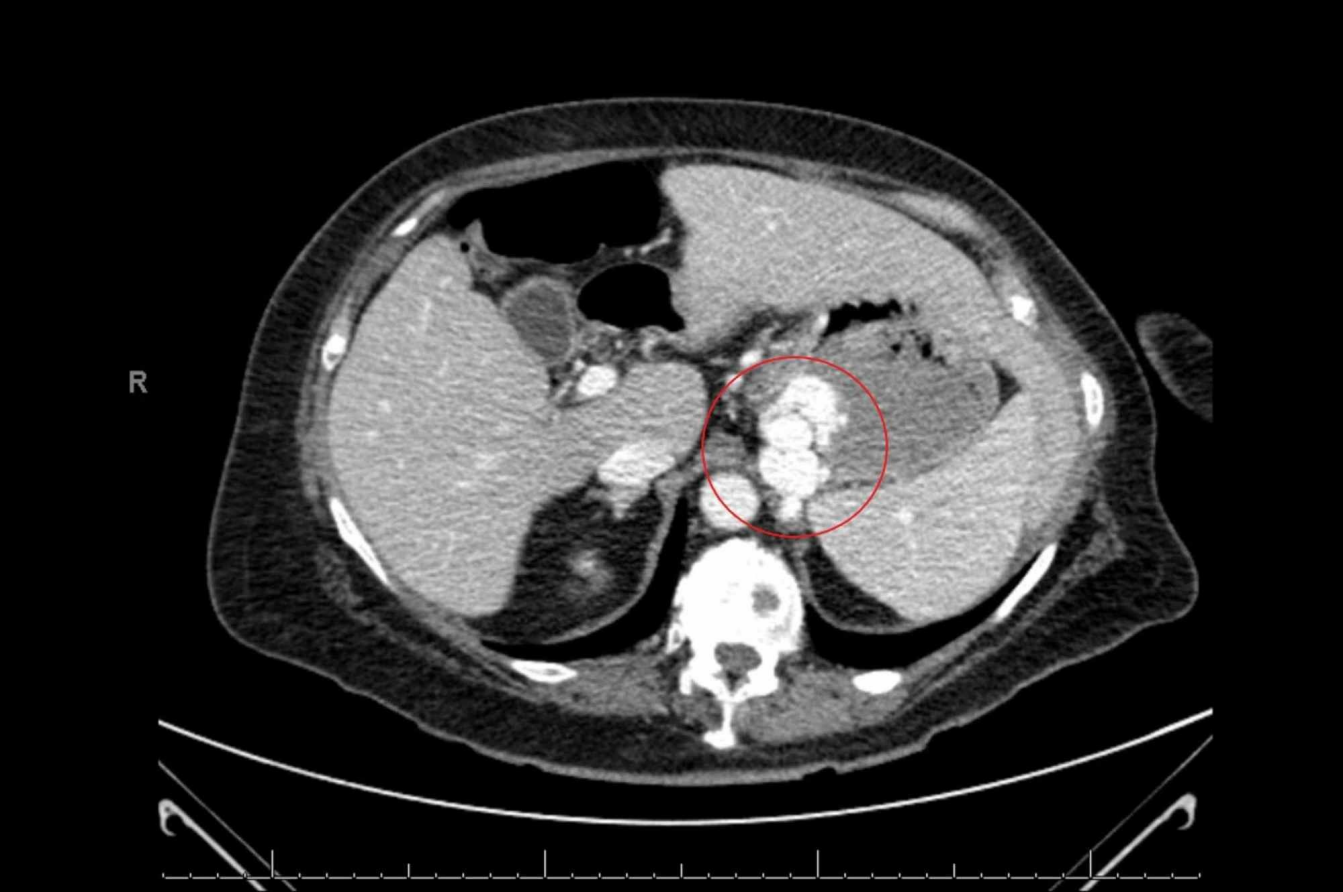
CT of abdomen/pelvis with IV oral contrast showing filling defect in the gastric cardia (highlighted)

**Figure 2:**
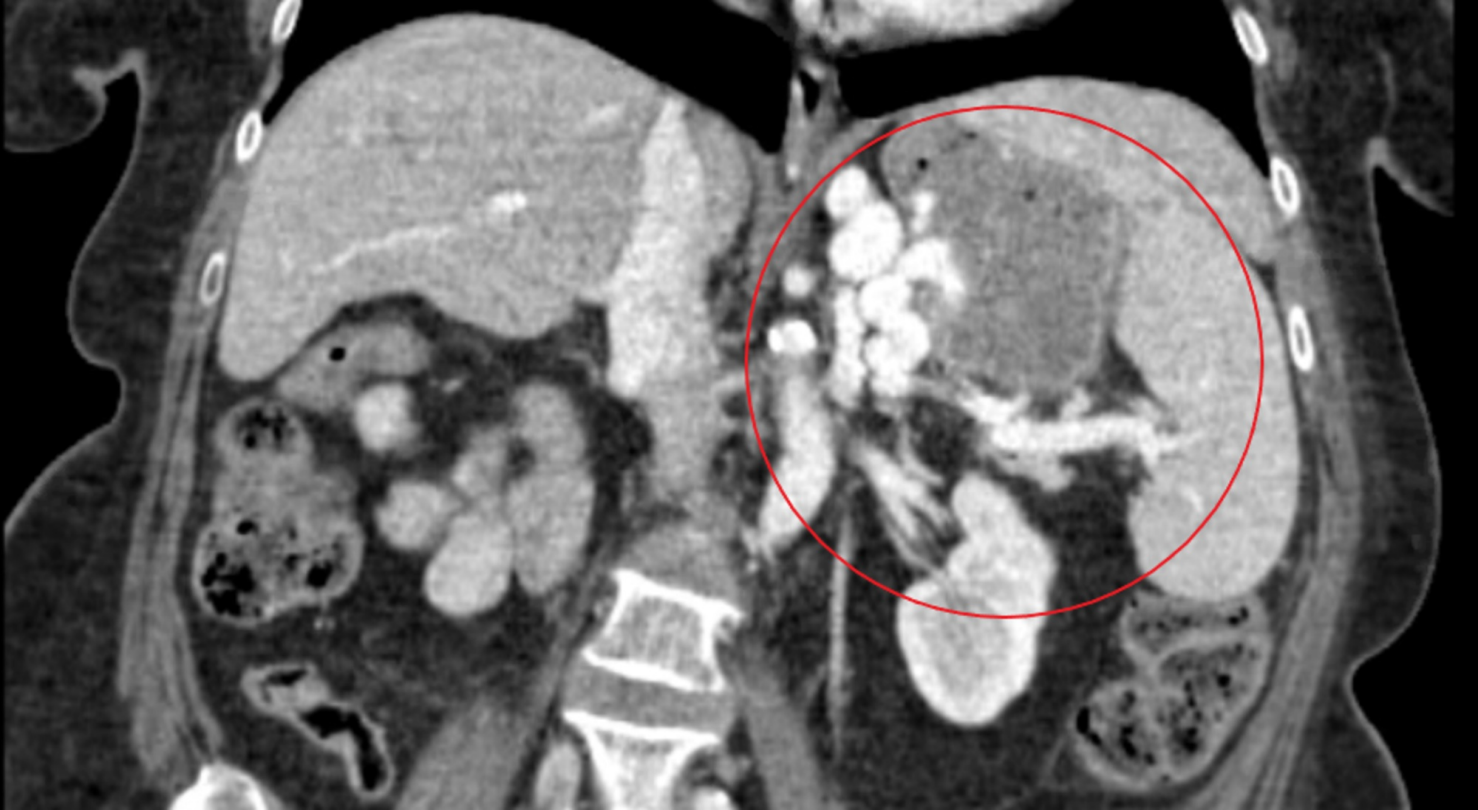
CT of abdomen/pelvis with IV oral contrast showing filling defect in the gastric cardia and splenic vein communication (highlighted)

Since both upper and lower GI endoscopies were consistently negative, we considered the possibility of doing either a capsule endoscopy or a Computed Tomography (CT) angiogram. However, considering the patient’s ongoing abdominal pain, a CT abdomen was done, which showed a lobulated mass in the gastric cardia that was suspicious for a solitary gastric varix (Figure 1, 2).

**Figure 3:**
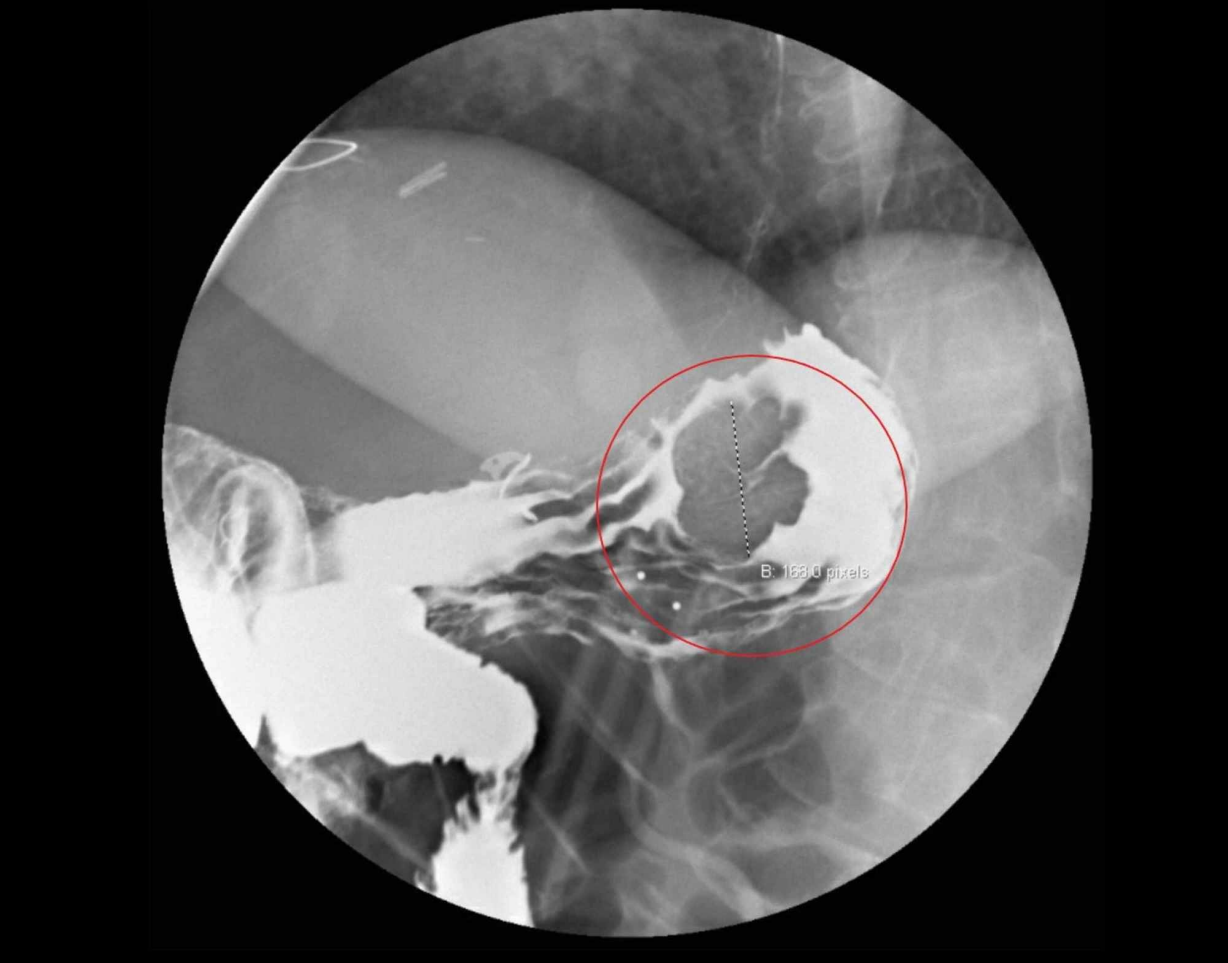
Upper gastrointestinal follow-through series showing gastric varix

**Figure 4:**
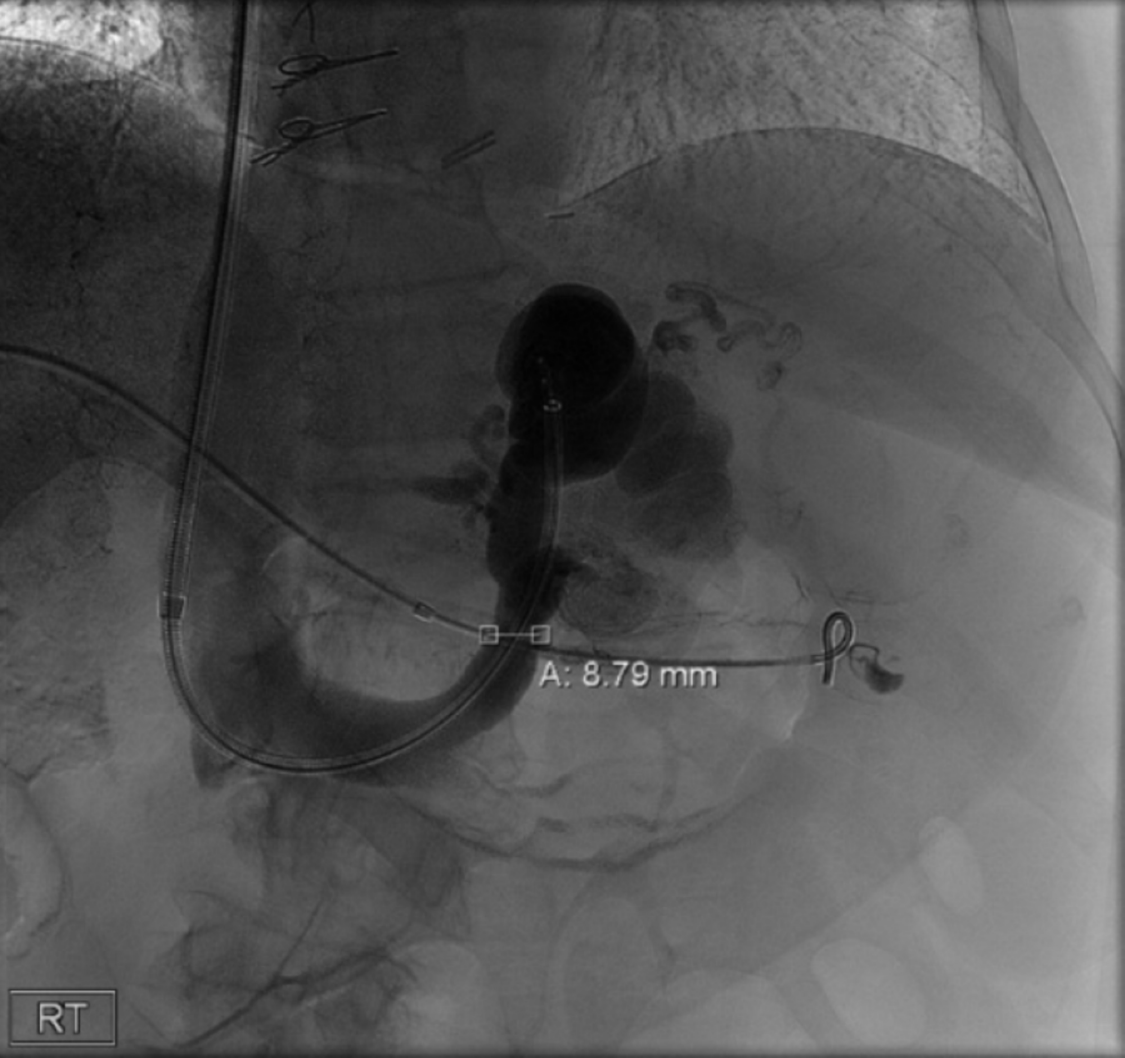
Venogram obtained during balloon-occluded retrograde transvenous obliteration demonstrating gastric varix

To confirm the mass-like defect noted on the CT of the abdomen, an upper GI and small bowel follow-through series was performed. The lobulated mass-like filling defect in the gastric cardia was consistent with a solitary gastric varix with an abnormal fold pattern (rosette) at the gastric cardia (Figure 3). Once she was deemed hemodynamically stable, she was transferred to an advanced GI center for interventional radiology guided embolization of varix. She subsequently underwent balloon-occluded retrograde transvenous obliteration (BRTO) which confirmed large varix emanating from the mid-splenic vein and connecting through gastric varix into a spleen-o-renal shunt (Figure 4). This was completely obliterated. Post-embolization venogram demonstrated patency of the splenic vein. She was then discharged with close follow up, and she has since remained free of symptoms.

## Discussion

Gastric varices are dilated submucosal veins that can result in a life-threatening upper GI bleed. Cirrhosis with subsequent portal hypertension is the most important cause of gastroesophageal varices. It is estimated that nearly 50% of patients with liver cirrhosis have already developed varices at the time of diagnosis [5].

There are several classifications used to describe gastric varices - Sarin, Hashizome, and Arakawa - of which Sarin is most commonly used [6]⁠. The Sarin classification groups varices into one of four categories: gastroesophageal varices (GOV) type 1: extension of esophageal varices along the lesser curvature; GOV type 2: extension of esophageal varices along great curvature; isolated gastric varices (IGV) type 1: gastric varices limited to fundus and Isolated gastric varices; IGV type 2: gastric varices outside fundus [7]⁠. Based on Sarin’s classification, varix in our case was IGV type 1.

This case report describes a unique isolated solitary gastric varix that was concealed in an abnormal fold in the gastric lining of the fundus and consequently was not visualized using conventional evaluation through EGD. Upper GI endoscopy is regarded as the diagnostic modality of choice for upper GI varices, with a reported sensitivity and specificity of 92-98% and 33-100% respectively [8]⁠. Current guidelines recommend an EGD within 12 hours for the diagnosis and management of a suspected variceal bleed; there are no specific recommendations regarding the application of non-endoscopic diagnostic modalities [5]. However, endoscopy can miss up to 10% of lesions if they are within reach of the scope, and 14% if they are not [9]⁠. In this case, the varix was identified using CT imaging, which is a non-invasive imaging modality. This prompted us to question whether CT imaging could be an alternative/adjunctive modality for evaluation of varices as it is relatively cost-effective and non-invasive. Yu Jen Tseng et al. conducted a meta-analysis evaluating the diagnostic performance of CT for gastroesophageal varices (GOV) [10]⁠. They determined the pooled sensitivity and specificity for identifying gastric varices (GV) were 0.955 (95% CI, 0.903-0.980) and 0.658 (95% CI, 0.433-0.829), respectively, with an Area Under the Receiver Operating Characteristics (AUROC) of 0.95, suggesting that CT imaging is a reasonable diagnostic strategy in cases with a high clinical suspicion for upper GI bleeding with a non diagnostic endoscopy. Other diagnostic approaches to upper GI bleeding include radionuclide imaging using 99 mTC-radionuclide tagged red blood cells (RBC), which carries a sensitivity and specificity of 93% and 95% respectively, angiography, wireless capsule endoscopy, and magnetic resonance angiography [8]⁠.

Hemorrhage from gastric varices can be managed with endoscopic and radiological modalities. Common endoscopy techniques include sclerotherapy, band ligation, obturation using cyanoacrylate tissue glue or thrombin injections depending upon the type of gastric varices [11]⁠. Of these, injection sclerotherapy has been largely abandoned due to high re-bleeding rates. Radiological interventions include transjugular intrahepatic portosystemic shunt (TIPS) and BRTO [12]⁠. These methods are adopted when adequate control of bleeding is not achieved with one of the endoscopic approaches or expertise to perform obturation is not available in Non-GEV 1 gastric varices.

BRTO is an endovascular technique which involves occlusion of the outflow veins of the portosystemic shunt, such as a gastrorenal shunt. This occlusion is achieved by the injection of a sclerosing agent into varix. Even though it has been common practice for the treatment of gastric varices in Asia over the last two decades, BRTO has been gaining more popularity in the US lately [13].

Some studies have shown that in comparison with TIPS, it is less invasive and can still be performed on patients with a poor hepatic reserve and hepatic encephalopathy [14]⁠. One of the complications associated with BRTO, although rare, is the reflux of the sclerosant agent into the portal or systemic vasculature [15]⁠. It has a high hemostasis rate ranging between 76.9 to 100% and a low risk of rebleeding [16]⁠. Overall, it is considered a reasonable therapeutic alternative to TIPS in the management of a gastric variceal bleed.

## Conclusions

Variceal hemorrhage is a major cause of upper GI bleeding in cirrhotic patients. Upper endoscopy is the diagnostic modality of choice for identifying varices, however, it is important to be cognizant about its limitations and consider the use of alternative diagnostic studies which can assist in determining the source of bleed in atypical presentations such as the case highlighted here.
